# Acute urinary retention due to corpus cavernosum penile metastasis from lung adenocarcinoma after targeted therapy: a case report and review of the literature

**DOI:** 10.3389/fonc.2024.1278245

**Published:** 2024-03-01

**Authors:** Wei Yan, Hui Fu, Huiqun Liu, Zhentian Liu, Xueliang Qi, Tanxiu Chen

**Affiliations:** ^1^ Department of Thoracic Oncology, Jiangxi Cancer Hospital, The Second Affiliated Hospital of Nanchang Medical College, Jiangxi Cancer Institute, Nanchang, China; ^2^ Department of Pathology, Jiangxi Cancer Hospital, The Second Affiliated Hospital of Nanchang Medical College, Nanchang, China; ^3^ Department of Urology, Jiangxi Cancer Hospital, The Second Affiliated Hospital of Nanchang Medical College, Nanchang, China; ^4^ Institute of Neurology and Department of Neurology, Jiangxi Academy of Clinical Medical Sciences, The First Affiliated Hospital of Nanchang University, Nanchang, China; ^5^ Jiangxi Key Laboratory of Translational Cancer Research, Jiangxi Cancer Hospital, The Second Affiliated Hospital of Nanchang Medical College, Nanchang, China

**Keywords:** metastasis, penile, adenocarcinoma of lung, targeted therapy, case report

## Abstract

**Background:**

Metastasis in penile corpus cavernosum from adenocarcinoma of lung is a rare but fatal disease, which was reported in cases without series studies. It causes various clinical symptoms seriously affecting the quality of life.

**Case presentation:**

A 72-year-old male smoker patient, who had a history of adenocarcinoma of lung after targeted therapy 36 months before, was admitted to Jiangxi Cancer Hospital because of presenting with aggressive dysuria and penis pain for one hour. A Foley catheter was inserted into the patient’s bladder with difficulty. Immediately do a bladder puncture. Emergency pelvic computed tomography (CT): a soft tissue nodule of 1.1 cm×1.4 cm was found in the cavernous area of the middle part of the penis, and the proximal urethra was dilated with a wide diameter of about 1.5 cm. The diagnosis of metastatic lung adenocarcinoma from the primary was made by CT-guided biopsy.

**Conclusion:**

The penis may be a site of metastasis from primary lung cancer, especially for old patient. Metastasis to the penis usually indicates that the primary lung cancer is at an advanced stage and the prognosis is very poor. More research is necessary to understand the underlying mechanism of adenocarcinoma of lung metastasis.

## Introduction

Penile cancer is an uncommon disease, with an anticipated 36,068 incidences worldwide in 2020 (0.92 cases per 100,000 persons) (1). It is astonishing that metastasis to the penis is such an uncommon clinical condition given that the penis has a large and intricate vascular. There have been more than 500 cases of metastatic penile cancer reported since the first case reported in 1870 (2). The nearby genitourinary and pelvic organs, particularly the bladder, prostate, and rectosigmoid colon, which together account for almost 75% of all metastatic lesions, are the primary site of origin. 25% of penile metastases originates from extrapelvic sources (3). The ratio of primary lung cancer to metastatic penile cancer was 4% to 6.2% (4, 5). In this case report, we describe a patient who, 36 months after receiving targeted therapy, developed acute urine retention as the first symptom of primary lung cancer. The study is approved by the Ethics Committee of the Jiangxi Cancer Hospital. Additionally, the patient’s son also gave his signed, informed agreement for the information and images to be used in this article. The literature on penile metastases from primary lung cancer is also reviewed, and its clinical characteristics, diagnosis, treatment, and prognosis are discussed.

## Case presentation

**Figure 1:**
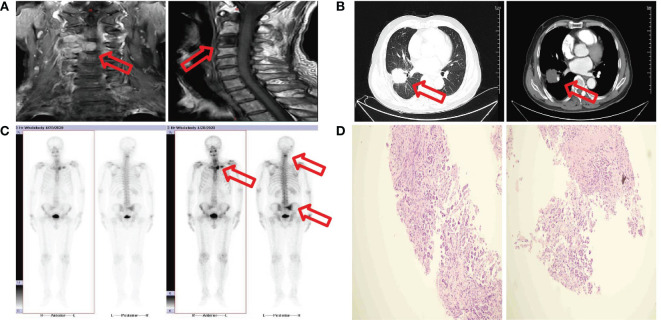
**(A)** MRI image shows C3/C4 vertebral bodies and their right attachments presented bone destruction with soft tissue mass. **(B)** CT image shows a mass measure 3.5 cm×3.9 cm in the right lower lobe. **(C)** Bone scan image shows multiple bone metastases. **(D)** Fine needle aspiration biopsy show adenocarcinoma.

Without any previous history of penile-related illness or family history of penile cancer, the patient, a 72-year-old male smoker, presented at our hospital (for dysuria with penile mild pain two months and acute urinary retention for one hour). The patient was admitted to Jiangxi cancer hospital on April 26, 2020 for the first time, because of neck pain for 8 months and higher symptoms for one day. C3/C4 vertebral bodies and their right attachments presented bone destruction with soft tissue mass by cervical spine MRI (**Figure 1A**). T1WI slightly low signal, T2WI slightly high signal, and DWI high signal (**Figure 1A**). Based on the MRI results, we considered tumor metastasis. Enhanced chest computed tomography revealed an irregular moderate enhancement soft tissue mass measuring 3.5 cm×3.9 cm in the right lower lobe of the lung with adjacent pleural thickening and pulling (**Figure 1B**). Multiple enhanced nodular shadows were observed in the mediastinum and right hilar lobe. The largest one, approximately 1.8 cm in diameter, was located in the right hilar area. Based on the enhanced chest computed tomography results, we considered lymph node metastasis. Additionally, osteolytic bone destruction was observed at the left edge of the sternum (**Figure 1B**). The radionuclide bone scan revealed multiple bone metastases, including 4th cervical vertebral body, the left sternoclavicular joint, sternum, sacrum, and the right sacroiliac joint (**Figure 1C**). Ultrasound doppler showed no obvious enlarged lymph nodes in the double neck or double clavicle. No abnormality was observed undergoing an electronic bronchoscopy. Laboratory examination revealed carcinoembryonic antigen (CEA) level was 54.47 ng/mL (reference value 0-5 ng/mL). Percutaneous lung fine needle aspiration pathology revealed adenocarcinoma (**Figure 1D**). The patient was diagnosed right lung adenocarcinoma with multiple bone metastasis. The stage was cT2aN2M1c phase IVc. The result of genetic test showed EGFR-del19 mutation. EGFR-TKIs are the preferential options for advanced non-small cell lung cancer (NSCLC) patients harboring EGFR mutations. Osimertinib was recommended as the standard first-line treatment for advanced or metastatic NSCLC patients with EGFR mutations. The patient received C3/C4 vertebral bodies metastatic site palliative radiation (DT: 30Gy/10F) on May 8, 2020, and was treated with Osimertinib (80mg po qd) on May 11, 2020. He had received treatment with “targeted” therapy for 3 years due to lung adenocarcinoma with multiple bone metastasis.

**Figure 2:**
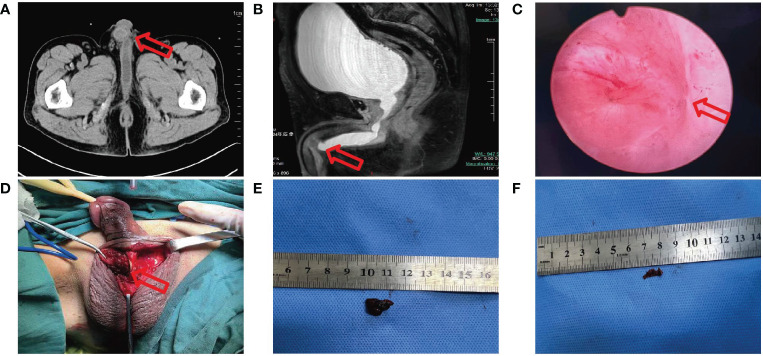
**(A)** CT image shows a mass measure 1.1 cm×1.4 cm in the middle cavernous body of penile with proximal urethral dilation. **(B)** MRI image shows a mass measure 1.2 cm×1.3 cm in the middle cavernous body of penile with local urethral stricture. **(C)** Electronic cystoscope image shows urethra was crushed. **(D-F)** During operation a fused nodule was found on the right side of the corpus cavernosum.

The patient was once again admitted to our hospital with the complaint of dysuria with penile mild pain for two months and acute urinary retention one hour on June 6, 2023. He was in good performance status (ECOG=1). However, it was difficult to insert a foley catheter into the patient’s bladder. Physical examination revealed a mass in the middle of penile measuring 1 cm×1.5 cm with ambiguous boundaries, poor activity, mild tenderness, no redness around, and normal skin temperature. We considered obstructive uropathy resulting in acute urinary retention, and immediately performed transabdominal cystostomy. A mass located in the middle cavernous body of penile measuring 1.1 cm×1.4 cm was detected by pelvic CT (**Figure 2A**). Local urethral stricture and proximal urethral dilation (widest diameter about 1.5 cm) were observed (**Figure 2A**). A heterogeneously enhancing mass located in the middle cavernous body of penile measuring 1.2 cm×1.3 cm was also detected by pelvic MRI (**Figure 2B**). Local urethral stricture and proximal urethral dilation were observed (**Figure 2B**). We considered that it may be a metastatic tumor. Fortunately, the patient’s head MRI did not reveal any abnormal changes. Laboratory examination revealed the total prostate specific antigen level was 0.778 ng/mL (reference value 0-4 ng/mL), and carcinoembryonic antigen (CEA) level was 46.51 ng/mL (reference value 0-5 ng/mL).

**Figure 3:**
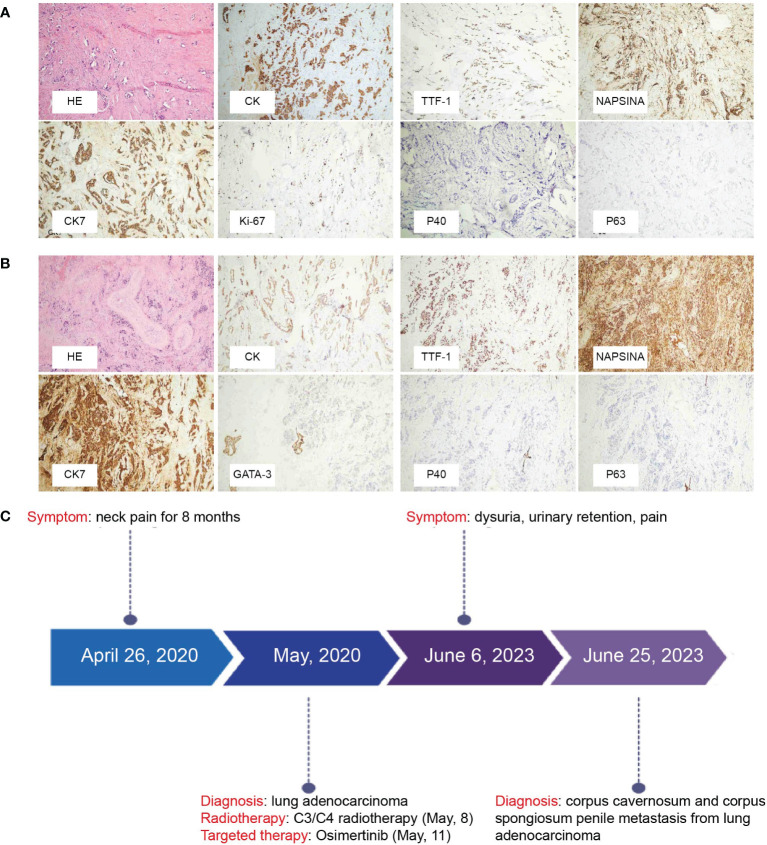
**(A)** Histopathological images of the nodule of corpus cavernosum penis show adenocarcinoma from primary lung cancer. H&E staining was performed. Immunohistochemical staining revealed that the tumor cells were positive for CK, TTF-1, NapsinA, CK7, Ki-67, and were negative for P40, and P63. Representative images are shown at ×200 magnification. **(B)** Histopathological images of the nodule of corpus spongiosum show adenocarcinoma from primary lung cancer. H&E staining was performed. Immunohistochemical staining revealed that the tumor cells were positive for CK, TTF-1, NapsinA, CK7, and were negative for GATA-3, P40, and P63. Representative images are shown at ×200 magnification. **(C)** Timeline of the patient’s diagnosis and treatment process.

Cytology of penile mass by fine needle aspiration guided by color ultrasound showed adenocarcinoma. Based on the patient’s previous lung cancer history, we highly suspected that the penile mass was of lung cancer origin. The anterior urethra was crushed and narrowed by the tumor when the electronic cystoscope enters the urethra at a 6 cm distance (**Figure 2C**). The patient underwent penile mass incisional biopsy on June 19, 2023. One fused nodule was found on the right of corpus cavernosum penis with urethral invasion (**Figures 2D-F**). The histological diagnosis of the nodule of corpus cavernosum penis was poorly differentiated adenocarcinoma with CK(+), TTF-1(+), NapsinA(+), CK7(+), Ki-67(+, 10%), P40 (-), P63 (-) (**Figure 3A**), and the histological diagnosis of the nodule of corpus cavernosum urethra was poorly differentiated adenocarcinoma with CK(+), TTF-1(+), NapsinA(+), CK7(+), GATA-3(-), P40(-), P63(-) (**Figure 3B**), which indicated that the penile metastasis from adenocarcinoma of lung. Currently, the patient was remaining on treatment (**Figure 3C**).

## Discussion

Lung cancer is the most common type of cancer worldwide, with an estimated 1.6 million fatalities per year (6). A set of histological subtypes generally known as NSCLC affect about 85% of patients; the two most prevalent subtypes are lung adenocarcinoma (LUAD) and lung squamous cell carcinoma (LUSC) (7). A major contributor to cancer-related deaths worldwide is still lung cancer. Non-small cell lung cancer (NSCLC) is still an incurable illness for the majority of patients despite numerous advancements in therapies over the past ten years. For many years, solely cytotoxic chemotherapy was the recommended course of treatment for advanced-stage NSCLC. However, the landscape of treatment has quickly changed with the introduction of targeted treatments and immunotherapy. The patient had a genetic mutation and suddenly developed acute urinary retention after 36 months of treatment with third-generation targeted drugs, and the pathology was identified as penile metastasis from primary lung adenocarcinoma. The case report was first diagnosed with acute urinary retention due to in corpus cavernosum penile metastasis from lung adenocarcinoma after three generations of targeted therapy.

The majority of malignant penis tumors are primary, with squamous cell carcinoma being the most common kind. Melanoma, basal cell carcinoma, and soft tissue sarcoma are rare but also occur (8). Since the penis has a complicated and rich vascular and lymphatic supply, it is rare for tumors to metastasize there (9). Penile metastasis from pulmonary carcinoma is even rarer. Regional lymph nodes, bone, brain, liver, adrenal gland, and pleura are frequent sites of metastasis for primary lung cancer. Primary lung cancer that has spread to the penis is typically regarded as end-stage and having a limited prognosis. Extrapulmonary symptoms are the initial indicator of diagnosis in 15% of individuals with primary lung cancer (10). The low incidence of the disease may be partially attributed to the fact that the penis is not routinely inspected (11). There is a need for more research to confirm the mechanism or molecular biology feature of penile metastasis of penile metastasis, particularly from primary lung cancer.

**Figure 4:**
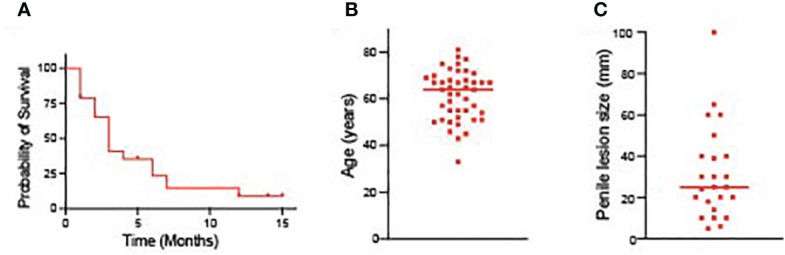
**(A)** The survival time of patients diagnosed with corpus cavernosum penile metastasis from lung adenocarcinoma. **(B)** The age of patients diagnosed with corpus cavernosum penile metastasis from lung adenocarcinoma. **(C)** Penile lesion size of patients diagnosed with corpus cavernosum penile metastasis from lung adenocarcinoma.

Lung cancer metastasizing to the penile is a rare occurrence, and the pathogenic mechanism is poorly understood. Such metastasis shows broad diffusion and is linked to a poor prognosis. It is difficult to estimate the exact incidence of this metastatic pattern because there aren’t many cases that have been documented. To address this, we conducted a thorough review of the literature, encompassing 44 previously reported cases along with our own case (**Table 1**). Among the reported cases, a total of 44 patients had lung cancer metastasizing to the peniles. The median survival time of these patients was 3 months (**Figure 4A**). Additionally, we generate two scatter plots in which the x axis is the patient diagnosed with corpus cavernosum penile metastasis from lung adenocarcinoma, and the y axis are the age and the size of penile lesion respectively. The median age of these patients was 64 years (**Figure 4B**). Penile lesion size reported ranged from 5 mm to as large as 100 mm. The median size of penile lesion was 25 mm (**Figure 4C**). In our case, metastasis was diagnosed at 36 months from the initial diagnosis of primary lung cancer.

**Table 1:** Summary of penile metastasis from primary lung cancer.

Study	Age (years)	Histology type	Metastatic site	Penile lesion size, mm	Clinical manifestations	Other distant Metastasis	Time of penile metastasis, M	Penis therapy	OS, M	References
1	33	EC	CC, CS	NR	UR, Priapism	Yes	NR	NR	<1	(12)
2	43	SCC	CC	NR	Mass, Priapism	Yes	NR	No	3	(12)
3	45	SCC	CC, CS, GP	NR	UR	Yes	NR	No	NR	(13)
4	46	SC	CC	NR	Priapism, Pain	No	ST	No	12	(14)
5	49	SCC	CC, CS	50	Mass	Yes	6	TTH	>15	(12)
6	50	SCC	CC	NR	Priapism	NR	NR	No	NR	(15)
7	50	SCC	CC, CS	30	Dysuria, pain	No	NR	CTH+RTH	4	(16)
8	51	SCC	CC	NR	Mass, Priapism	No	NR	CTH	NR	(17)
9	51	AC	CC, CS, GP	20	Dysuria, SP, Ulceration	Yes	ST	CTH	<2	(4)
10	51	AC	GP	NR	Pain, Ulceration	Yes	ST	CTH	3	(14)
11	52	SCC	CC, CS	100	Pollakisuria, BU	Yes	ST	No	1	(18)
12	54	LCC	CC	NR	Mass	Yes	NR	RTH	7	(19)
13	55	SCC	GP	25	Ulceration, Pain	No	ST	No	6	(10)
14	55	AC	CC, CS, GP	10	SP, Pain	Yes	ST	RTH	2	(12)
15	55	SCC	CC, CS	20	Mass	No	ST	CTH	6	(14)
16	57	SCC	CC	NR	Mass	No	ST	CTH	4	(3)
17	57	NSCLC	CC	20	ED, Mass	Yes	ST	RTH	NR	(20)
18	59	AC	CC	18	Mass	Yes	1	CTH+RTH	>5	(11)
19	60	AC	CC, CS, GP	NR	Priapism	Yes	ST	CTH	3	(21)
20	62	AC	CC, GP	24	Pain	Yes	ST	CTH	7	(2)
21	62	EHE	CC, CS	40	Mass, Pain	NO	7	CTH	2	(5)
22	64	SCLC	GP	NR	Mass	NR	NR	No	>12	(22)
23	64	ASC	CC	5	Pain	Yes	ST	CTH	>14	(14)
24	65	SCC	NR	NR	Mass	Yes	NR	CTH	3	(23)
25	65	SCC	CC, GP	NR	Priapism	Yes	ST	RTH	3	(24)
26	67	SCC	CC, CS, GP	65	Mass, UR	NO	36	CTH+RTH	6	(5)
27	67	SCC	CC	NR	SP	Yes	NR	No	<1	(25)
28	67	SCC	CC	30	Mass	Yes	24	No	<1	(26)
29	67	SCC	CC, CS	NR	Dysuria	Yes	6	No	2	(27)
30	67	SCC	Foreskin	6	Mass	No	ST	CTH	6	(28)
31	67	SCC	CC	30	Mass	YES	24	No	NR	(26)
32	68	SCC	CC, CS, GP	NR	UR, Priapism	YES	17	No	<1	(29)
33	68	SCC	CC, CS	NR	Priapism	No	ST	No	12	(14)
34	69	SCC	CC	NR	Pain, Priapism	NR	NR	No	3	(30)
35	70	SCC	CC, CS	NR	Pain, Priapism	YES	NR	No	2	(31)
36	71	ASC	CC	60	Mass	Yes	ST	CTH	7	(32)
37	72	AC	CC	NR	UR, Priapism	NR	NR	No	1	(33)
38	73	AC	CS	25	UR	NR	ST	NR	1	(34)
39	75	SCC	CC	60	Mass	YES	6	RTH	3	(35)
40	75	SCC	CC	10	Mass	No	ST	CTH	NR	(36)
41	77	SCLC	GP	10	Mass	Yes	6	CTH	3	(28)
42	78	SCC	CC	40	Mass	Yes	ST	No	<1	(37)
43	81	SCC	CC, CS	39	Priapism, Pain	Yes	ST	RTH	3	(14)
44	72	AC	CC, CS	14	Pain, Dysuria, UR	Yes	36	TTH	>1	Present case

AC, adenocarcinoma; ASC, adenosquamous carcinoma; BU, burning on urination; CC, corpora cavernosa; CS, corpus spongiosum; CTH, chemotherapy; EC, epithelial carcinoma; ED, erectile dysfunction; EHE, epithelioid hemangioendothelioma; GP, glans penis; LCC, large cell carcinoma; NR, not recorded; NSCLC, nonsmall cell lung cancer; OS, over survival time after penile metastasis; RTH, radiotherapy; SC, sarcomatoid carcinoma; SCC, squamous cell carcinoma; SCLC, small cell lung cancer; SP, swelling of penis; ST, same time; TTH, target therapy; UR, urinary retention. No. 44th is the patient we present, No. 1-43 from the literature.

It is still debatable how secondary penile cancer spreads. Due to its copious blood supply and status as an end organ in terms of the arterial, venous, and lymphatic systems, the rarity of metastatic involvement of the penis has been a clinical mystery. Most academics favor retrograde venous metastasis at this time, and the anatomical fact that the dorsal vein of the penis enters the prostate and bladder to form a pelvic venous plexus, which is characterized by low pressure, the absence of a venous valve, and a profusion of anastomotic branches, supports this theory. Primary tumor cells retrogradely enter the dorsal venous system of the penis through the vaginal vein system if the intra-abdominal pressure rises abruptly as a result of severe coughing, faeces, or obstruction of the venous return channel. This ultimately results in tumor metastasis. It was reported that the retrograde venous metastatic pathway was further substantiated by a cancer thrombus in the dorsal vein of the penis. In addition, the direct infiltration route seems to be another way that primary cancer cells can enter the bulbous urethra and travel directly along the perineal plane before causing the development of a secondary penile tumor (38). Other theories include cancer metastasis caused by tumor cells entering the urethra and nosocomial transmission, such as cystoscopy biopsy and transurethral prostate and bladder resection. Another theory is that cancer cells may migrate via the nerves to the penis (39). Usually, penile metastasis or direct tumour infiltration of the corpora cavernosa cause priapism linked to solid tumours. Three to eight percent of priapism instances are caused by neoplasias, and in eighty percent of cases, the primary tumour has a genito-urinary origin (40). This point of view is further supported by the case report that describes the secondary malignant penile erection in renal cell carcinoma caused by paraneoplastic leukaemia response (41).

Although primary lung cancer incidence rises in older populations and there is also an increasing trend in young persons, the median age of diagnosis is 60.5 ± 10.7 years (42). Clinical signs of penile metastases can take many different forms. The most frequent clinical symptom of pulmonary cancer is a penile nodule, which is typically found in elderly people. Around 20% of patients reported penile pain and discomfort, and some may have trouble peeing due to urine retention (43). There is an intriguing situation where the cause of erectile dysfunction remains unclear (20). The mass is typically found in the shaft of the penis, less frequently in the head or foreskin. The underlying cause could be that the corpora cavernosa interact readily due to an inadequate midline septum.

Nearly one-third of all penile metastases are often found concurrently with the underlying tumor, whereas the remaining two-thirds are found some months after the primary tumor is found (44). Apart from the initial lung cancer, the penis is the only site of metastasis in approximately one-third of patients (27.5%). As previously said, the initial stages to properly detecting penile metastases include a strong clinical suspicion, a thorough anamnesis, and a physical examination. In addition to physical examination, imaging tests (non-invasive modalities) like CT, MRI, PET, and ultrasound scans can be extremely helpful in clinical diagnosis and disease staging. The irregular mass was represented on the CT scan with mild-to-moderate amplification, demonstrating the anatomical link between the cancer lesions and the surrounding organs (45). A tumor’s size, depth, location of malignant invasion, and extent of damage to neighbouring tissues can all be identified by MRI. These lesions typically have modest signal strength on T1-weighted imaging, much like the corpora cavernosa around them. They appear non-homogenous on T2-weighted imaging, with low to intermediate signal intensities that are easily recognized against the high background intensity of the corpora cavernosa (46). Despite its high cost, PET/CT can be utilized to detect metastatic foci. The gold standard for diagnosis and a tool for separating primary tumors from metastases is histopathological analysis.

The standard of care has not been established since there are so few instances of secondary penile tumors. The commonly used treatment methods in clinic were as follows: local therapy (surgery and radiation therapy) and systemic therapy (chemotherapy, targeted therapy, and immunotherapy), or a combination of these treatments. The primary lung cancer’s histological type, the size, the location, the number of metastatic tumors, the patient’s age, and general health status all have a role in determining the course of treatment for penile metastatic cancer. Patients with these illnesses should receive palliative care in light of their poor prognosis in order to reduce or eliminate their unbearable symptoms. Therapy for pain and anxiety is also necessary, and if necessary, parenteral opioids and/or an anxiolytic should be used. Palliative local resection or radiation therapy can reduce discomfort and enhance quality of life (9, 27, 35).

## Conclusion

In summary, metastasis to the penis from primary lung cancer is extremely rare, the penis may be a site of metastasis from primary lung cancer especially for those older men who with a genetic mutation and survive for a long time. Penile metastasis from primary lung cancer in most cases is a lethal pathology that indicates wide dissemination of oncological disease and has a very poor prognosis. Since primary lung cancer remains the leading cause of cancer-related deaths worldwide and since the prolongation of survival in lung cancer patients, more cases of penile metastasis might be detected in the future. Early detection, then appropriate management of penile metastasis will be more important. When metastases are limited to the penis, surgery yields the greatest results. In order to improve the prognosis for lung adenocarcinoma with penile metastases, further study on cancer is required.

## Data availability statement

The raw data supporting the conclusions of this article will be made available by the authors, without undue reservation.

## Ethics statement

The studies involving humans were approved by The Institutional Review Board of the Institute of Jiangxi cancer hospital. The studies were conducted in accordance with the local legislation and institutional requirements. The human samples used in this study were acquired from a by- product of routine care or industry. Written informed consent for participation was not required from the participants or the participants’ legal guardians/next of kin in accordance with the national legislation and institutional requirements. Written informed consent was obtained from the individual(s) for the publication of any potentially identifiable images or data included in this article.

## Author contributions

WY: Conceptualization, Data curation, Formal analysis, Software, Supervision, Writing – original draft, Investigation, Validation, Writing – review & editing. HF: Data curation, Formal analysis, Validation, Writing – review & editing, Resources. HL: Formal analysis, Resources, Validation, Writing – review & editing, Supervision. ZL: Formal analysis, Resources, Supervision, Validation, Writing – review & editing. XQ: Formal analysis, Resources, Supervision, Validation, Writing – review & editing. TC: Formal analysis, Supervision, Conceptualization, Data curation, Funding acquisition, Software, Writing – original draft.
